# Contributions of *MYOC* and *CYP1B1* mutations to JOAG

**Published:** 2008-03-13

**Authors:** Behnaz Bayat, Shahin Yazdani, Afagh Alavi, Mohsen Chiani, Fereshteh Chitsazian, Betsabeh Khoramian Tusi, Fatemeh Suri, Mehrnaz Narooie-Nejhad, Mohammad H Sanati, Elahe Elahi

**Affiliations:** 1 National Institute of Genetic Engineering and Biotechnology, Tehran, Iran; 2 Ophthalmic Research Center, Shaheed Beheshti University of Medical Sciences, Tehran, Iran; 3 School of Biology, University College of Science, University of Tehran, Tehran, Iran; 4 Research Center for Gastroenterology and Liver Diseases, Shaheed Beheshti University of Medical Sciences, Tehran, Iran; 5 Center of Excellence in Biomathematics, School of Mathematics, Statistics and Computer Science, University of Tehran, Tehran, Iran

## Abstract

**Purpose:**

To investigate the role of *MYOC* and *CYP1B1* in Iranian juvenile open angle glaucoma (JOAG) patients.

**Methods:**

Twenty-three JOAG probands, their available affected and unaffected family members, and 100 ethnically matched control individuals without history of ocular disease were recruited. Clinical examinations of the probands included slit lamp biomicroscopy, intraocular pressure (IOP) measurement, gonioscopic evaluation, fundus examination, and perimetry measurement. Familial cases were classified according to the mode of inheritance. Exons of *MYOC* and *CYP1B1* were sequenced, and novel variations assessed in the control individuals. Potential disease-associated variations were tested for segregation with disease status in available family members.

**Results:**

The mode of inheritance of the disease in the families of four probands (17.4%) appeared to be autosomal dominant and in at least eight (34.8%) to be autosomal recessive. Four patients carried *MYOC* mutations, and an equal number carried *CYP1B1* mutations. The *MYOC* mutations were heterozygous; two of them (p.C8X and p.L334P) are novel, and one codes for the shortest truncated protein so far reported. Autosomal recessive inheritance was consistent with inheritance observed in families of patients carrying *CYP1B1* mutations. All these patients carried homozygous mutations.

**Conclusions:**

*MYOC* and *CYP1B1* contributed equally to the disease status of the Iranian JOAG patients studied. The contribution of the two genes appeared to be independent in that no patient carried mutations in both genes. The fraction of Iranian patients carrying *MYOC* mutations was comparable to previously reported populations.

## Introduction

Glaucoma is a heterogeneous group of optic neuropathies, which manifest by optic nerve head cupping or degeneration of the optic nerve, resulting in a specific pattern of visual field loss [1-3]. Increased intraocular pressure (IOP) is often associated with the condition. If not treated by medical or surgical therapy in time, glaucoma leads to irreversible visual field loss and, ultimately, blindness. Therefore, diagnosis at an early stage of the disease is very important. The disease affects approximately 65 million people worldwide and is considered the second leading cause of blindness [4]. Glaucoma in some families demonstrates Mendelian inheritance. It is sub-grouped into three major classes on the basis of etiology, anatomy of the anterior chamber, and age of onset [1]. Primary open-angle glaucoma (POAG; OMIM 137760) accounts for 70% of glaucoma cases in Caucasian populations and usually affects individuals past the age of 40 [5]. In this form of glaucoma, the anterior chamber angle and the trabecular meshwork appear normal. It is associated with variable severity and phenotypic expressivity [6,7]. POAG is sometimes divided into the two sub-classes of adult-onset and juvenile-onset, the latter appearing between early childhood and the age of 40 [7]. The more rare juvenile form (JOAG) has been reported to usually exhibit autosomal dominant inheritance. Clinical features of the juvenile form are generally more severe [6].

Although many loci have been reported for POAG, (GLC1A to GLC1N; Human Gene Nomenclature), genes for only three have been identified [2]. The three genes code for myocilin (*MYOC*; GLC1A; OMIM 601652), optineurin (*OPTN*; GLC1E; OMIM 602432), and WD repeat containing protein 36 (*WDR36*; GLC1G; OMIM 609669) [7-9]. The functions of these genes in the eye are not known. Myocilin is a bipartite protein, containing a myosin-like NH_2_-terminal domain and an olfactomedin homology COOH-terminal domain [10]. Most of disease-associated mutations in *MYOC* affect the olfactomedin-like domain. Haploinsufficiency does not appear to be the primary disease mechanism of *MYOC* mutations [11]. Several studies have indicated that mutant forms are associated with a gain of function or negative dominant effect [10,12-15]. Mutations in *MYOC* have been found in sporadic cases and in patients inheriting the disease in an autosomal dominant fashion, most often in those with juvenile onset [6,7,16,17]. There is evidence that interactions between different genes may cause glaucoma in some individuals [18-22].

*CYP1B1* (OMIM 601771), encoding cytochrome P4501B1, is a gene commonly associated with primary congenital glaucoma (PCG; OMIM 231300); however, mutations in *CYP1B1* have been reported in JOAG patients [23-27]. Some patients carrying *CYP1B1* mutations also carried *MYOC* mutations and the etiology of disease in these was considered digenic. The pathway by which *CYP1B1* causes glaucoma is not understood [26,27]. PCG is characterized by an anatomic defect of the trabecular meshwork (trabeculodysgenesis) and an age of onset in the neonatal or infantile period.

The genetic basis of PCG among Iranian patients has recently been studied, and it was found that nearly 70% of Iranian PCG patients carry disease-associated mutations in *CYP1B1* [28]. In other populations, mutations in *CYP1B1* account for the disease status of 20%–100% of PCG patients [29]. A heterogeneous range of mutations were identified among the Iranian patients, many of which were novel. Here, we assess the association of *MYOC* and *CYP1B1* in a cohort of 23 Iranians affected with JOAG. Phenotypic features of patients carrying mutations in these genes are presented.

**Figure 1:**
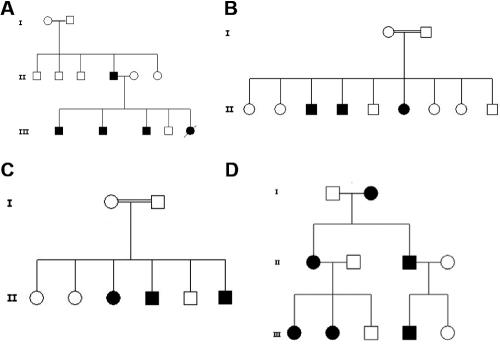
Iranian JOAG pedigrees. (**A**) JG 102: Inheritance in this pedigree does not appear to be conclusively autosomal dominant because individuals of generation I are unaffected. Autosomal recessive inheritance seemed possible because of consanguinity and extensive inbreeding in village of residence. (**B**) JG 103 and (**C**) JG 111: Autosomal recessive inheritance is suggested in these two pedigrees because of multiple affected siblings born to unaffected parents who are first cousins. (**D**) JG 118: Autosomal dominant inheritance is suggested in this pedigree because affected individuals are observed in three consecutive generations. The affected individuals of the last two generations each had only one affected parent.

## Methods

This research was performed in accordance with the Declaration of Helsinki and with the approval of the Ethics Board of the International Institute of Genetic Engineering and Biotechnology in Iran. The participants or authorized family members all consented to participate after being informed of the nature of the research. Twenty-three unrelated JOAG patients were recruited from the ophthalmic division of the Labafi-Nejhad Hospital (associated with Shahid Beheshti University of Medical Sciences and Health Services) in Tehran. The hospital is a national reference center and patients from throughout Iran are referred to it. All patients were diagnosed by one of the authors (S.Y.) who is a glaucoma specialist. Slit lamp biomicroscopy, IOP measurement, gonioscopic evaluation of the angle, fundus examination, and measurement of perimetry were performed whenever possible. IOP measurements were obtained using Goldmann tonometry. Criteria for diagnosis were the presence of at least two of these criteria: an IOP greater than 21 mmHg in at least one eye or intereye IOP asymmetry exceeding 8 mmHg; characteristic glaucomatous optic nerve head or retinal nerve fiber layer (RNFL) changes (e.g., vertical cupping, neural rim thinning or loss, RNFL dropout); and visual field defects not attributable to other causes. All patients presented with an open anterior chamber angle in the affected eyes. Patients with other ocular or systemic anomalies were excluded. Classification of juvenile onset POAG was based on the age of diagnosis, ranging between the age of 10 and 40 years. One-hundred ethnically matched, unrelated control individuals were also recruited from those older than 60 years of age and without self-reported familial history of ocular diseases.

**Table 1:** Mode of JOAG inheritance in families of patients.

**Sporadic cases**	**Familal cases**			**Mode of Inheritance of familial cases**		
	**Consanguious parents**	**>1 Affected individual**	**Consanguious parents & >1 affected individual**	**Autosomal dominant**	**Autosomal recessive**	**Unknown**
JG104	JG107	JG102	JG100	JG113	JG101	JG100
JG120		JG113	JG101	JG117	JG103	JG102
JG123		JG114	JG103	JG118	JG105	JG110
JG127		JG117	JG105	JG121	JG107	JG112
JG130		JG121	JG110		JG111	JG122
		JG122	JG111		JG114	JG134
		JG129	JG112		JG129	
		JG134	JG118		JG131	
			JG131			

Sporadic cases are indicated in the left-most column. The next three columns separate the familial cases according to criteria by which they were classified as familial. The three right-most columns indicate mode of inheritance in each of the familial cases.

The three exons of *MYOC* were amplified by polymerase chain reaction (PCR; primer sequences available upon request). All PCR products were sequenced in both forward and reverse directions with the same primers used in the PCRs, using the ABI big dye chemistry and an ABI Prism 3700 instrument (Applied Biosystems, Foster City, CA). Sequences were analyzed by comparing them to *MYOC* reference sequences (NT_004487, NM_000261, and NP_000252) using the Sequencher software (Gene Codes Corp., Ann Arbor, MI). To determine segregation of found variations with disease status in families, appropriate restriction enzyme digestion reactions were set up and restriction fragment length polymorphism (RFLP) was performed. Similarly, the presence of novel variations in control individuals was assessed by RFLP. Predicted effects of variant sequences on splicing were determined by comparison with known canonical splice site motifs at the splice site prediction by neural network site. To assess the extent of conservation of a novel variation in *MYOC* thought to be associated with disease, the amino acid sequences of 12 myocilin proteins from as many species were obtained from SwissProt and aligned using the ClustalW software (European Bioinformatics Institute, Hinxton, UK). Core haplotypes defined by three common *MYOC* intragenic polymorphisms were assessed in the probands using PLINK.

For mutation analysis of *CYP1B1*, coding exons 2 and 3 of *CYP1B1* were sequenced and the sequences were analyzed as already reported [28]. Exon 1 of *CYP1B1* was not analyzed because no disease-associated variations have been found among the many PCG patients screened from various populations [28,29].

## Results

The clinical features of the sporadic JOAG patients and the probands of the familial cases are presented in Table 2. Average age of onset of the patients was 21.3 years old.

**Table 2:** Phenotypic features of Iranian JOAG patients.

**Pedigree**	**F/S**	**Inheritance**	**Age at diagnosis**	**C/D ratio R/L**	**IOP max (mmHg) R/L**	**Trabeculotomy**	**Age of onset range**	**MYOC Haplotype**
JG104#	S		14 years	0.3/0.6	30/34	1x		H1, H2*
JG120	S		40 years	0.6/0.6	40/40	1x		H1, H1*
JG123#	S		17 years	0.3/0.4	22/29	1x		H1, H1*
JG127	S		32 years	0.3/0.3	30/30	1x		H1, H1*
JG130#	S		14 years	0.3/0.5	20/30	1x		H1, H2*
JG113	F	AD	40 years	0.5/0.9		multiple	5–40 years	H2, H3
JG117	F	AD	17 years	0.9/0.4	34/34	1x	17–22 years	H2, H3
JG118	F	AD	17 years		33/33	2x	Birth-60 years	H1, H1*
JG121	F	AD	27 years	0.3/0.4	33/34	1x	27–58 years	H1, H2*
JG101	F	AR	16 years	0.6/0.3	33/34	2x	Birth-22 years	H1, H2*
JG103##	F	AR	19 years	0.5/0.5	/33	1x	19–20 years	H1, H3 or H2, H4
JG105	F	AR	24 years		normal/33	1x	24–29 years	H2, H2*
JG107	F	AR	10 years	0.3/0.3		1x		H2, H2*
JG111	F	AR	32 years		high/high	2x	32–36 years	H1, H2*
JG114##	F	AR	18 years	0.3/0.3		1x	5–30 years	H3, H3*
JG129##	F	AR	L: birth R: 12 years	0.6/0.3	30/		Birth-12 years	H1, H1*
JG131	F	AR	L: 12 years R: birth	0.5/0.3	28/24	1x	Birth-45 years	H1, H4
JG100##	F	?	10 years	0.5/0.6	35/35	3x	Birth-12 years	H1, H2*
JG102#	F	?	19 years	0.5/0.3	33/34	1x	7–20 years	H2, H2*
JG110	F	?	14 years	0.5/0.3		2x	14–35 years	H2, H2*
JG112	F	?	30 years	0.5/0.5		1x	5–60 years	H1, H1*
JG122	F	?	25 years	0.3/0.3		1x		H1, H4
JG134	F	?	30 years	0.4/0.9	34/34			H1, H4

Unknown clinical features are left blank. For familial cases, features of the proband of the families are presented. The meaning of each symbol and abbreviation is given in the following: F/S indicated the familial/sporadic status of patients: F, familial; S, sporadic; AD: autosomal dominant; AR: autosoamal recessive; ?: unknown mode of inheritance; R: right eye; L: left eye; Age of onset range is the range of age of onset in other affected family members; *MYOC* haplotypes are defined by SNPs at positions c.-83, c.227, and IVS2+35 in the same order. H1: -GGG-, H2: -GGA-, H3: -AAA-, H4: -AAG-; The sharp (hash mark) indicates patients in whom *MYOC* disease-associated variations were found; the double sharp indicates patients in whom *CYP1B1* disease-associated variations were found; the asterisk indicates that the haplotypes of these patients were unambiguous. Those of the remaining patients were predicted.

The patients were recruited consecutively without regard to familial status of disease. Patients were designated sporadic if they reported no consanguinity between parents and no other incidence of disease among relatives. If the previous criteria did not apply, they were designated familial. The mode of inheritance before mutation analysis in some familial cases was not straightforward and these were indicated as unknown. Parent to child inheritance was not necessarily an indication of autosomal dominant inheritance because of extensive inbreeding in some pedigrees, and reported non-consanguinity may have been misleading as all individuals within a pedigree sometimes belonged to small isolated villages where consanguineous marriages were common. Classification was further hampered because of possible incomplete penetrance of both *CYP1B1* and *MYOC* mutations [24,30-32]. An example of a pedigree (JG 102) in which the mode of inheritance was initially difficult to establish is presented in Figure 1A. For families where the mode of JOAG inheritance was clear, it was designated as autosomal recessive or autosomal dominant. The designations are presented in Table 1, and some pedigrees are shown in Figure 1.

Seven sequence variations were identified in the *MYOC* gene of the Iranian JOAG patients (Table 3). Four of the variations, c.-83G>A, c.227G>A, IVS2+35G>A, and c.975G>A are single nucleotide polymorphisms (SNPs) previously reported not to be associated with disease [11,16,33,34]. The first three SNPs were common among our cohort of patients and were found in both the heterozygous and homozygous states. C.975G>A was observed in only one patient in the heterozygous state. Core intragenic SNP haplotypes defined by the three common variants were assessed for the two *MYOC* alleles of all the patients (Table 2). Seventeen of the probands were homozygous at all or heterozygous at most at one of these variant positions, and their haplotypes could therefore be defined unambiguously Four haplotypes, –GGG- (H1), -GGA- (H2),–AAA- (H3), and –AAG- (H4), that are defined by nucleotides at positions c.-83, c.227, and IVS2+35 were observed. H1 (47.7%) and H2 (36.4%) were by far the most common haplotypes in our cohort of patients.

**Table 3:** *MYOC* variations in Iranian JOAG patients.

Gene location*	**cDNA** location*#	**Exon**	**Intron**	**Effect** **on** protein*	**Number of patients** **(pedigree IDs)**		**Total** **number** **variant allele**	**Frequency** **of variant** **allele**	**Reference** **SNP** **number**
					**Heterozygous**	**Homozygous**			
g.-83G>A	c.-83G>A	1			7 (103, 113, 117,119, 122,131,134)	1 (114)	9	19.57%	rs2075648
**g.46C>A##**	**c.24C>A##**	1		**p.C8X##**	2 (123, 130)	0	2	4.35%	
g.249G>A	c.227G>A	1		R76K	7 (103, 113, 117, 119, 122, 131, 134)	1 (114)	9	19.57%	rs2234926
g.14072G>A	IVS2+35G>A		2		6 (100, 101, 103,111, 121, 130)	8 (102, 104, 105, 107, 110, 113, 114, 117)	22	47.83%	rs2032555
g.16169G>A	c.975G>A	3		T325T	1 (100)	0	1	2.17%	
**g.16195T>C##**	**c.1001T>C##**	3		**L334P##**	1 (102)	0	1	2.17%	
**g.16303C>T**	**c.1109C>T**	3		**P370L**	1 (104)	0	1	2.17%	

The asterisk shows that the reference sequences used were NT_004487, NM_000261, and, NP_000252. Variations thought to cause JOAG are shown in bold. The meaning of each symbol is given in the following: the sharp (hash mark) indicates that the “A” of the initiation codon was designated +1; the double sharp indicates a novel mutation.

**Figure 2:**
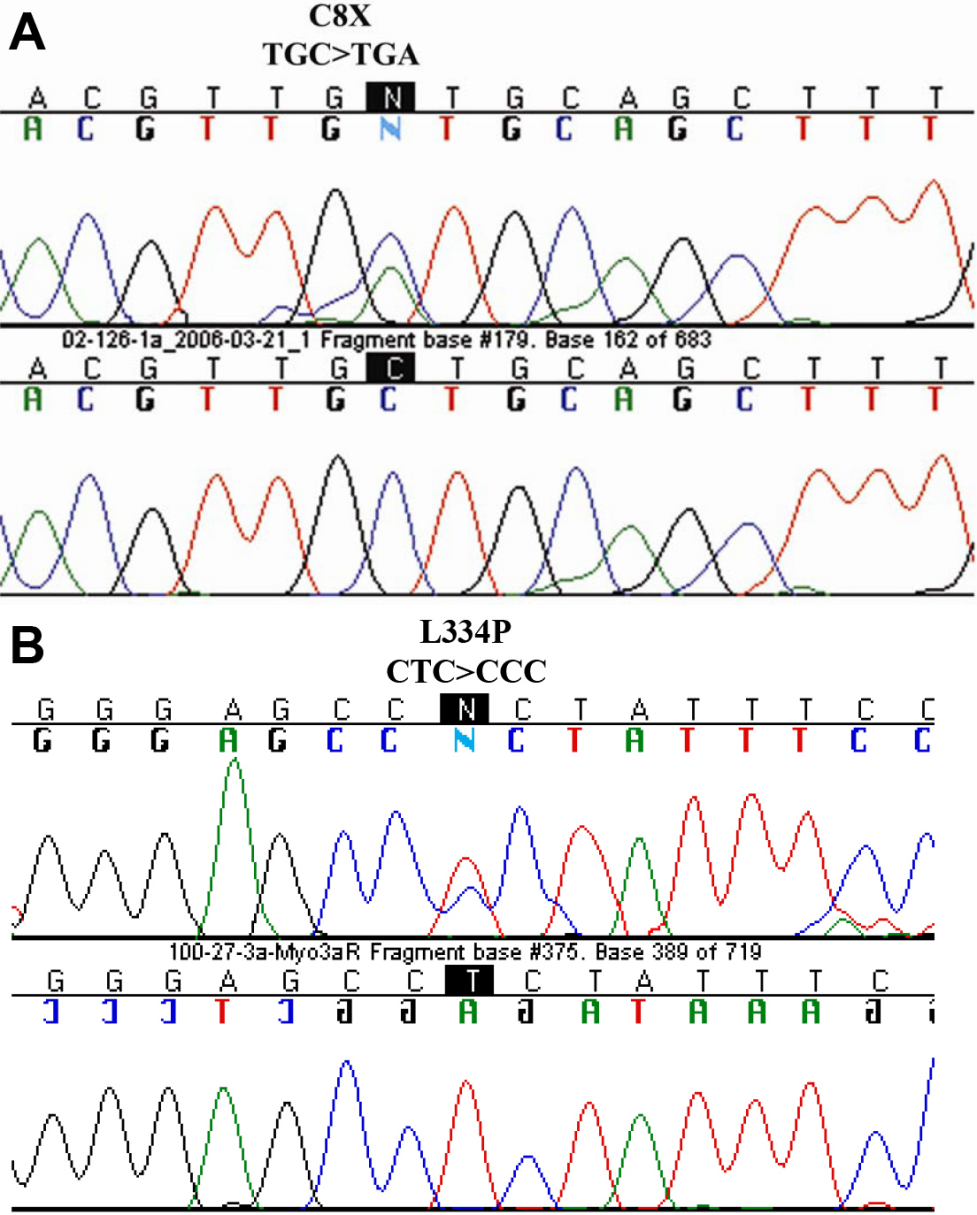
Novel mutations in *MYOC*. **A:** At the top, the sequence chromatogram is showing the heterozygous mutation, c.24C>A, which causes the codon change UGC to UGA, resulting in C8X; at the bottom, the chromatograph shows the wild type sequence. **B**: The top sequence chromatogram shows the heterozygous mutation, c.1001T>C, which is causing codon change CUU to CCU, resulting in L334P; the bottom chromatograph illustrates the wild type sequence.

C.24C>A and c.1001T>C are novel sequence variations in *MYOC* (Figure 2). C.24C>A, observed in the heterozygous state in two patients (JG 123 and JG 130), creates a stop codon early in the coding sequence after the seventh amino acid residue (C8X). It is thought to be associated with JOAG because it was found in two unrelated sporadic JOAG patients, shown to be absent by RFLP in the DNA of three available unaffected family members, and shown to be absent by RFLP in 100 control individuals (not shown). Identity by descent of the C8X mutation in the two patients could not be established as it was associated on haplotype H1 in one (JG 123) and on haplotype H1 or H2 in the other (JG 130). C.1001T>C, which results in L334P in the olfactomedin domain, is also thought to be associated with disease status. It results in an amino acid alteration at a position completely conserved in the myocilin protein of 12 species, including species as distally related as human and zebrafish (Table 4; bold, underlined amino acid). The variation was found in one patient (JG 102) in the heterozygous state on haplotype background H2. It was shown by RFLP to segregate with disease status among available members of this family (Figure 1A). Additionally, it was not observed by RFLP in the DNA of 100 ethnically matched control individuals (not shown). Age of diagnosis of the four affected family members was between 7 and 20 years old. The variation c.1109C>T resulting in P370L in the olfactomedin domain, was found in one sporadic case (JG 104) in the heterozygous state on haplotype H2. This variation has previously been reported as a disease-associated variation in several populations [34-36]. It is likely to be the cause of JOAG in patient JG 104. Unfortunately, other family members were not available for genetic analysis.

Four of the Iranian JOAG patients carried disease-associated mutations in the *CYP1B1* gene. All four carried the mutations in the homozygous state. C.182G>A (g. 3987G>A), causing G61E, was observed in three of the JOAG patients (JG 100, JG 103, and JG 114), and c. 1103G>A (g. 7940G>A), causing R368H, was observed in one patient (JG 129). The patient carrying R368H was affected with PCG in one eye (onset at birth) and with JOAG in the other. G61E and R368H have previously been reported to cause PCG in patients of various ethnicities, and they are common mutations among Iranian PCG patients [28]. The mode of inheritance in the pedigrees of three of the JOAG patients (JG 103, JG 129, and JG 114) was clearly autosomal recessive (Table 1). Affected siblings from only one of the pedigrees (JG 103) were available for genetic analysis, and it was shown by RFLP that they all carried homozygous *CYP1B1* mutations (Figure 1B). Unfortunately, unaffected siblings in the four pedigrees with *CYP1B1* mutations were not available for genetic analysis. However, some of the 14 unaffected siblings are expected to be heterozygous on a statistical basis. Similarly, the parents of three of the probands, all of whom are expected to harbor at least one mutated *CYP1B1* allele, were self-reported not to be affected with an ocular disorder. It has been reported that parents of PCG patients, many of whom carried the same G61E mutation found in three of our patients, are not at increased risk of developing glaucoma [30]. The father in the fourth pedigree (JG 100) was affected, but he was an offspring of a consanguineous marriage in a highly inbred pedigree, making it possible that he too was homozygous for the *CYP1B1* mutation. There was no obvious difference between age at diagnosis and phenotypic features of patients with *MYOC* and *CYP1B1* mutations.

## Discussion

Our cohort of JOAG patients had two notable features. One is that of the patients whose disease was classified as familial, four belonged to pedigrees in which the mode of inheritance of disease was autosomal dominant while eight belonged to pedigrees with an autosomal recessive mode of inheritance (Table 1). Although autosomal recessive inheritance of JOAG has been reported, the inheritance of the disease for familial cases is generally considered to be autosomal dominant [1,6,36,37]. JOAG is caused by more than one gene, and some of the genes may exert their effect in a recessive fashion. The role of these genes is less likely to be discerned in genetic studies on populations where consanguineous marriages are infrequent, and most of the studies on JOAG have been done on such populations [35]. The relatively high rate of inbreeding within the Iranian population is expected to allow mutations in these putative genes to show their phenotypic effects. It would be of interest to establish whether autosomal recessive inheritance of JOAG is common in other populations where consanguineous marriages are common such as the population of Saudi Arabia.

Another interesting feature of our cohort is that among the 17 families in which more than one individual was affected, one member in five of the families (29.4%) was affected from the time of birth and could be classified as a case of primary congenital glaucoma (Table 2). The preponderance of this situation in multi-case families supports the proposal made by other investigators that the disturbance of common biochemical pathways may be involved in the etiology of PCG and JOAG [20,24,38]. The observations of PCG (onset at birth) in one eye and JOAG (onset at age of 12) in the other eye of two probands (JG 129 and JG 131) further support this conjecture.

A mutation in *MYOC* was assessed to be the cause of JOAG in 4 of the 23 probands (17.4%) screened. This figure falls within the range reported for other populations [6,16,17]. C.24C>A that causes C8X was a novel disease-associated variation observed in two unrelated patients. The phenotypic features of the two patients, including age at diagnosis, cup to disc ratio, and IOP, were quite similar (Table 2). The protein product of the mutated allele is expected to be the shortest truncated MYOC reported to date. In addition to causing early protein truncation during protein synthesis, the early nonsense codon may also result in reduced levels of mRNA [39]. Previously, mutation Arg46X was reported in patients from Korea, Japan, and China [17,33,36], all countries in the Far East. After initial association of Arg46X and several other variations in *MYOC* with glaucoma, the causative nucleotide changes were also identified in control individuals [33,40-42]. The absence of a tight association between the variations and disease phenotype is a reflection of the complexity of the etiology of glaucoma [35]. With regards to C8X, the causative mutation was absent in the DNA of three unaffected siblings (age 25–28 years old) and 100 control individuals. On the other hand, at least one parent of each of the probands is expected to have harbored the variation. Both parents of both probands were reported by the probands not to have any form of ocular disorder, suggesting incomplete penetrance. Unfortunately, the parents were not available for genetic analysis. C8X may accompany a normal phenotype within specific genetic and/or environmental backgrounds, and an assessment of its penetrance must await studies in larger cohorts of affected and normal controls. If indeed C8X can cause JOAG as it appears to do in the probands of JG 123 and JG 130, this raises questions with regards to a gain of function or negative dominant effect model of disease causation by *MYOC* mutations [10,12,13]. Either the mRNA or the very short truncated protein product of the mutated allele can exert the same effect, or other models such as haploinsufficiency must be considered for at least some of the variations [12,13,43].

In addition to C8X, L334P was a novel disease-associated variation found among the Iranian pedigrees. Given that approximately 70 glaucoma-associated variations have been identified in *MYOC* among the many patients screened in the 10 years since the discovery of the gene, finding two novel mutations among only 23 probands is notable. This finding testifies to the high heterogeneity of the Iranian population and the potential value of this population for genetic studies [28,43,44]. With respect to *MYOC*, no single mutation has yet been observed in Caucasian, African, and Asian populations, and many mutations have been found in only specific regions [35,45-47]. As C8X was found in two unrelated Iranian patients and not found in other countries, it may have originated in Iran and so far have spread only to individuals within this region. *MYOC* mutations are generally considered relatively recent in human history [35]. C8X occurred on intragenic haplotype H1 and possibly H2, and L334P occurred on intragenic haplotype H2 (see Table 2). In case these variations are found in the future in other populations in association with different haplotypes, the observation will be an indication of a recurrence of mutation.

**Table 4:** Alignment of L334P in myocilin proteins.

Gene	L334P	Seq ID
**MYOC_*Homo sapiens***	**GAVVYSGS L YFQGAES**	NP_000252.1
MYOC_*Canis lupus familiaris*	GAVVYRGS **L** YFQGAGS	NP_001041495
MYOC_*Canis familiaris*	GAVVYRGS **L** YFQGAGS	Q2PT31
MYOC_*Felis catus*	GAVVYWGS **L** YFQGAES	AAS68633.1
MYOC_*Pan troglodytes*	GAVVYSGS **L** YFQGAES	XP_513995.2
MYOC_*Macaca fascicularis*	GAVVYSGN **L** YFQGAES	AAO40254.1
MYOC_*Oryctolagus cuniculus*	GAVVYAGS **L** YFQGAGS	NP_001075619
MYOC_*Mus musculus*	GAVVYAGS **L** YFQGAES	NP_034995.2
MYOC_*Rattus norvegicus*	GAVVYSGS **L** YFQGAES	NP_110492.1
MYOC_*Sus scrofa*	GAVVYQGS **L** YFQGASS	NP_999151.1
MYOC_*Bos taurus*	GAVVYRGS **L** YFQAAES	BAA77298.1
MYOC_*Danio rerio* (Zebrafish)	GATMYKGS **L** YYQRRLS	Q5F0G5

Sequence ID numbers are from the expasy server and NCBI.

P370L is one of the few *MYOC* mutations likely to have occurred more than once in human history [48]. The mutation in the Iranian proband is associated with haplotype H2, but available information does not allow us to ascertain whether it shares ancestry with previously reported P370L alleles or represents an independent mutation event.

Homozygous mutations in *CYP1B1* were assessed to be the cause of JOAG in 4 of the 23 probands (17.4%) screened, the same number as those whose disease was caused by *MYOC*. Mutations in *MYOC* were not observed in any of these four patients, and none of the individuals in our cohort were observed to carry only a single mutated *CYP1B1* allele. Patients affected with primary congenital or open angle glaucoma carrying double heterozygous mutations, one in CYP1B1 and the other in *MYOC*, have previously been reported in three studies [20,24,49]. The authors suggested that *CYP1B1* may act as a modifier locus for *MYOC* in promoting open angle glaucoma. In another study, among a large cohort of 236 French Caucasian POAG patients (with the median age of 48 years at diagnosis), 12 individuals (5.1%) carrying *CYP1B1* mutations were identified [21]. Only one *CYP1B1* mutated allele was found in 10 of them, and a *MYOC* mutation was not found in any of the 12 patients. It was suggested that a modifying locus other than *MYOC* or environmental factors may be involved in the disease status of the patients carrying heterozygous *CYP1B1* mutations. Similarly, in a recent study on 200 POAG patients from India, *CYP1B1* mutations were identified in nine patients (4.5%) and only one of these nine patients carried two mutated alleles [23]. This JOAG patient was reported not to harbor mutations in *MYOC* and *OPTN*, and it was suggested that *CYP1B1* was the primary cause of his disease status. Finally, in a study on 82 Spanish POAG patients, nine persons (10.9%) were reported to carry one putative disease-causing *CYP1B1* allele; none carried two mutated alleles [25]. Both *CYP1B1* mutations observed in the homozygous state in the Iranian patients were among the heterozygous mutations observed in previous studies [23,25].

Compared to these studies, the observation that *CYP1B1* appears to be the cause of JOAG in a larger proportion of the patients (17.4%) may not be significant because of our small sample size. However, it may reflect a higher frequency of mutated *CYP1B1* alleles in the Iranian gene pool; *CYP1B1* has a role in a relatively large percentage of Iranian PCG patients (70% as compared to 50% for French and 40% for Indian patients) [28].

With respect to JOAG being associated with one or two mutated *CYP1B1* alleles, it appears to be more often associated with two alleles in the Iranian population. Of course, it is possible that in a larger cohort, some JOAG patients with only one *CYP1B1* mutated allele would be found. It may be that individuals carrying one mutated *CYP1B1* allele will be only mildly symptomatic and therefore less likely to be identified.

In conclusion, our results suggest that *MYOC* and *CYP1B1* are each important in the etiology of JOAG, at least among Iranians. Precluding possible effects of common SNPs in the two genes, each appears to be able to promote disease independently, and therefore, both *MYOC* and *CYP1B1* should be considered potential primary causes of JOAG. It is thus recommended that both genes be screened for mutations in individuals at risk, particularly in relatives of glaucoma-affected individuals known to harbor mutations in these genes. Non-interventionist treatments are likely to be effective if applied at early stages of disease progression and could help prevent irreversible visual impairment.

## References

[1] Ray K, Mukhopadhyay A, Acharya M (2003). Recent advances in molecular genetics of glaucoma.. Mol Cell Biochem.

[2] Fan BJ, Wang DY, Lam DSC, Pang CP (2006). Gene mapping for primary open angle glaucoma.. Clin Biochem.

[3] Sarfarazi M (1997). Recent advances in molecular genetics of glaucomas.. Hum Mol Genet.

[4] Quigley HA (1996). Number of people with glaucoma worldwide.. Br J Ophthalmol.

[5] Shields MB, Ritch R, Krupin T. Classification of the glaucomas. In: Ritch R, Shields MB, Krupin T, editors. The Glaucomas. St. Louis (MO):Mosby-Year Book. P. 717–25.

[6] Wiggs JL, Lynch S, Ynagi G, Maselli M, Auguste J, Del Bono EA, Olson LM, Haines IL (2004). A genomewide scan identifies novel early-onset primary open-angle glaucoma loci on 9q22 and 20p12.. Am J Hum Genet.

[7] Stone EM, Fingert JH, Alward WLM, Nguyen TD, Polansky JR, Sunden SLF, Nishimura D, Clark AF, Nystuen A, Nichols BE, Mackey DA, Ritch R, Kalenak JW, Craven ER, Sheffield VC (1997). Identification of a gene that causes primary open angle glaucoma.. Science.

[8] Rezaie T, Child A, Hitchings R, Brice G, Miller L, Coca-Prados M, Heon E, Krupin T, Ritch R, Kreutzer D, Crick RP, Sarfarazi M (2002). Adult-onset primary open angle glaucoma caused by mutations in optineurin.. Science.

[9] Monemi S, Spaeth G, DaSilva A, DaSilva G, Popinchalk S, Ilitchev E, Liebmann J, Ritch R, Heon E, Crick RP, Child A, Sarfarazi M (2005). Identification of a novel adult-onset primary open-angle glaucoma (POAG) gene on 5q22.1. Hum Mol Genet.

[10] Tamm ER (2002). Myocilin and glaucoma: facts and ideas.. Prog Retin Eye Res.

[11] Fingert JH, Stone EM, Sheffield VC, Alward WL (2002). Myocilin glaucoma.. Surv Ophthalmol.

[12] Morissette J, Clepet C, Moisan S, Dubois S, Winstall E, Vermeeren D, Nguyen TD, Polansky JR, Cote G, Anctil JL, Amyot M, Plante M, Falardeau P, Raymond V (1998). Homozygotes carrying an autosomoal dominant TIGR mutation do not manifest glaucoma.. Nat Genet.

[13] Kim BS, Savinova OV, Reedy MV, Lun Y, Gan L, Martin J, Tomarev SI (2001). ohn SWM, Johnson RL. Targeted disruption of the myocilin gene (Myoc) suggests that human glaucoma –causing mutations are gain of function.. Mol Cell Biol.

[14] Fautsch MP, Bahler CK, Jewison DJ, Johnson DH (2000). Recombinant TIGR/MYOC increases outflow resistance in the human anterior segment.. Invest Ophthalmol Vis Sci.

[15] Shepard AR, Jacobson N, Millar JC, Pang IH, Steely HT, Searby CC, Sheffield VC, Stone EM, Clark AF (2007). Glaucoma-causing myocilin mutants require the Peroxisomal targeting signal-1 receptor (PTS1R) to elevate intraocular pressure.. Hum Mol Genet.

[16] Alward WL, Fingert JH, Coote MA, Johnson AT, Lerner SF, Junqua D, Durcan FJ, McCarthy PJ, Mackey DA, Sheffield VC, Stone EM (1998). Clinical features associated with mutations in the chromosome 1 open-angle glaucoma gene (GLC1A).. N Engl J Med.

[17] Fingert JH, Héon E, Liebmann JM, Yamamoto T, Craig JE, Rait JL, Kawase K, Hoh S-T, Buys YM, Dickinson J, Hockey RR, Williams-Lynn D, Trope G, Kitazawa Y, Ritch R, Mackey DA, Alward WLM, Sheffield VC, Stone EM (1999). Analysis of myocilin mutations in 1703 glaucoma patients from five different populations.. Hum Mol Genet.

[18] Copin B, Brezin AP, Valtot F, Dascotte JC, Bechetoille A, Garchon HJ (2002). Apolipoprotein e-promoter single-nucleotide polymorphisms affect the phenotype of primary open-angle glaucoma and demonstrate interaction with the myocilin gene.. Am J Hum Genet.

[19] Craig JE, Baird PN, Healey DL, McNaught AI, McCartney PJ, Rait JL, Dickinson JL, Roe L, Fingert JH, Stone EM, Mackey DA (2001). Evidence for genetic heterogeneity within eight glaucoma families, with the glc1a gln368stop mutation being an important phenotypic modifier.. Ophthalmology.

[20] Vincent AL, Billingsley G, Buys Y, Levin AV, Priston M, Trope G, Williams-Lyn D, Heon E (2002). Digenic inheritance of early-onset glaucoma: Cyp1b1, a potential modifier gene.. Am J Hum Genet.

[21] Melki R, Colomb E, Lefort N, Brézin AP, Garchon HJ (2004). CYP1B1 mutations in French patients with early-onset primary open-angle glaucoma.. J Med Genet.

[22] Baird PN, Foote SJ, Mackey DA, Craig J, Speed TP, Bureau A (2005). Evidence for a novel glaucoma locus at chromosome 3p21–22.. Hum Genet.

[23] Acharya M, Mookherjee S, Bhattacharjee A, Bandyopadhyay AK, Daulat Thakur SK, Bhaduri G, Sen A, Ray K (2006). Primary role of CYP1B1 in Indian juvenile-onset POAG patients.. Mol Vis.

[24] Chakrabarti S, Kaur K, Komatireddy S, Acharya M, Devi KR, Mukhopadhyay A, Mandal AK, Hasnain SE, Chandrasekhar G, Thomas R, Ray K (2005). Gln48His is the prevalent myocilin mutation in primary open angle and primary congenital glaucoma phenotypes in India.. Mol Vis.

[25] López-Garrido M-P, Sánchez-Sánchez F, López-Martínez F, Aroca-Aguilar J-D, Blanco-Marchite C, Coca-Prados M (2006). Escribano.Heterozygous *CYP1B1* gene mutations in Spanish patients with primary open-angle glaucoma.. Mol Vis.

[26] Nebert DW, Russell DW (2002). Clinical importance of the cytochromes P450.. Lancet.

[27] Stoilov I, Jansson I, Sarfarazi M, Schenkman JB (2001). Roles of cytochromes P450 in development.. Drug Metabol Drug Interact.

[28] Chitsazian F, Tusi BK, Elahi E, Saroei HA, Sanati MH, Yazdani S, Pakravan M, Nilforooshan N, Eslami Y, Mehrjerdi MA, Zareei R, Jabbarvand M, Abdolahi A, Lasheyee AR, Etemadi A, Bayat B, Sadeghi M, Banoei MM, Ghafarzadeh B, Rohani MR, Rismanchian A, Thorstenson Y, Sarfaraz M (2007). CYP1B1 mutation profile of Iranian primary congenital glaucoma patients and associated haplotypes.. J Mol Diagn.

[29] Sarfarazi M, Stoilov I, Schenkman JB (2003). Geneticsa and biochemistry of primary congenital glaucoma.. Ophthalmol Clin North Am.

[30] Bejjani BA, Stockton DW, Lewis RA, Tomey KF, Dueker DK, Jabak M, Astle WF, Lupski JR (2000). Multiple CYp1B1 mutations and incomplete penetrance in an inbred population segregating primary congenital glaucoma suggest frequent de novo events and adominant modifier locus.. Hum Mol Genet.

[31] Povoa CA, Malta RFS, Rezende MM, de Melo KFS, Giannella-Neto D (2006). Correlation between genotype and phenotype in primary open angle glaucoma of Brazilian families with mutations in exon 3 of the TIGR/MYOC gene.. Arq Bras Oftalmol.

[32] Ramprasad VL, Sripriya S, Ronnie G, Nancarrow D, Saxena S, Hemamalini A, Kumar D, Vijaya L, Kumaramanickavel G (2005). Genetic homogeneity for inherited congenital microcoria loci in an Asian Indian pedigree.. Mol Vis.

[33] Pang CP, Leung YF, Fan B, Baum L, Tong WC, Lee WS, Chua JK, Fan DS, Liu Y, Lam DS (2002). TIGR/MYOC gene sequence alterations in individuals with and without primary open- angle glaucoma.. Invest Ophthalmol Vis Sci.

[34] Hewitt AW, Mackey DA, Craig JE (2008). Myocilin allele-specific glaucoma phenotype database.. Hum Mutat.

[35] Gong G, Kosoko-Lasaki O, Haynatzki GR, Wilson MR (2004). Genetic dissection of myocilin glaucoma.. Hum Mol Genet.

[36] Yoon SJ, Kim HS, Moon JI, Lim JM, Joo CK (1999). Mutations of the *TIGR/MYOC* Gene in Primary Open-Angle Glaucoma in Korea. 1999. Am J Hum Genet.

[37] Johnson AT, Drack AV, Kwitek AE, Cannon RL, Stone EM, Alward WL (1993). Clinical features and linkage analysis of a family with autosomal dominant juvenile glaucoma.. Ophthalmology.

[38] Zhuo YH, Wang M, Wei YT, Huang YL, Ge J (2006). Analysis of MYOC gene mutation in a Chinese glaucoma family with primary open-angle glaucoma and primary congenital glaucoma.. Chin Med J (Engl).

[39] Strubin M, Bert C, Mach B (1986). Alternative splicing and alternative initiation of translation explain the four forms of the Ia antigen-associated invariant chain.. EMBO J.

[40] Mabuchi F, Yamagata Z, Kashiwagi K, Tang S, Iijima H, Tsukahara S (2001). Analysis of myocilin gene mutations in Japanese patients with normal tension glaucoma and primary open-angle glaucoma.. Clin Genet.

[41] Jansson M, Marknell T, Tomic L, Larsson LI, Wadelius C (2003). Allelic variants in the MYOC/TIGR gene in patients with primary open-angle, exfoliative glaucoma and unaffected controls.. Ophthalmic Genet.

[42] Wiggs JL, Vollrath D (2001). Molecular and clinical evaluation of patient hemizygous for TIGR/MYOC.. Arch Ophthal.

[43] Elahi E, Khodadad A, Kupershmidt I, Ghasemi F, Alinasab B, Naghizadeh R, Eason RG, Amini M, Esmaili M, Esmaeili Dooki MR, Sanati MH, Davis RW, Ronaghi M, Thorstenson YR (2006). A haplotype framework for cystic fibrosis mutations in Iran.. J Mol Diagn.

[44] Alavi A, Elahi E, Tehrani MH, Amoli FA, Javadi MA, Rafati N, Chiani M, Banihosseini SS, Bayat B, Kalhor R, Amini SS (2007). Four mutations (three novel, one founder) in *TACSTD2* among Iranian GDLD Patients.. Invest Ophthalmol Vis Sci.

[45] Brézin AP, Adam MF, Belmouden A, Lureau M-A, Chaventré A, Copin B, Gomez L, Dupont de Dinechin S, Berkani M, Valtot F, Rouland J-F, Dascotte J-C, Bach J-F, Garchon H-J (1998). Founder effect in GLC1A-linked familial open-angle glaucoma in Northern France.. Am J Med Genet.

[46] Angius A, Eliseo De Gioia E, Loi A, Fossarello M, Sole G, Orzalesi N, Grignolo F, Cao A, Pirastu M (1998). A novel Mutation in the *GLC1A* Gene Causes Juvenile Open-angle Glaucoma in 4 Families From the Italian Region of Puglia.. Arch Ophthalmol.

[47] Vasconcellos JP, Melo MB, Costa VP, Tsukumo DM, Basseres DS, Bordin S, Saad ST, Costa FF (2000). Novel mutation in the MYOC gene in primary open angle glaucoma patients.. J Med Genet.

[48] Adam MF, Belmouden A, Binisti P, Brezin AP, Valtot F, Bechetoille A, Dascotte JC, Copin B, Gomez L, Chaventre A, Bach JF, Garchon HJ (1997). Recurrent mutations in a single exon encoding the evolutionarily conserved olfactomedin-homology domain of TIGR in familial open-angle glaucoma.. Hum Mol Genet.

[49] Kaur K, Reddy AB, Mukhopadhyay A, Mandal AK, Hasnain SE, Ray K, Thomas R, Balasubramanian D, Chakrabarti S (2005). Myocilin gene implicated in primary congenital glaucoma.. Clin Genet.

